# Combined Analysis of Extant Rhynchonellida (Brachiopoda) using Morphological and Molecular Data

**DOI:** 10.1093/sysbio/syx049

**Published:** 2017-05-08

**Authors:** David W. Bapst, Holly A. Schreiber, Sandra J. Carlson

**Affiliations:** *Department of Earth and Planetary Sciences, University of California, Davis, One Shields Avenue, Davis, CA 95616, USA*; *Penn Dixie Fossil Park and Nature Reserve, 3556 Lakeshore Rd, Ste. 210 Blasdell, NY 14219, USA*

**Keywords:** Brachiopoda, combined analyses, morphology, paleontology, phylogenetics

## Abstract

Independent molecular and morphological phylogenetic analyses have often produced discordant results for certain groups which, for fossil-rich groups, raises the possibility that morphological data might mislead in those groups for which we depend upon morphology the most. Rhynchonellide brachiopods, with more than 500 extinct genera but only 19 extant genera represented today, provide an opportunity to explore the factors that produce contentious phylogenetic signal across datasets, as previous phylogenetic hypotheses generated from molecular sequence data bear little agreement with those constructed using morphological characters. Using a revised matrix of 66 morphological characters, and published ribosomal DNA sequences, we performed a series of combined phylogenetic analyses to identify conflicting phylogenetic signals. We completed a series of parsimony-based and Bayesian analyses, varying the data used, the taxa included, and the models used in the Bayesian analyses. We also performed simulation-based sensitivity analyses to assess whether the small size of the morphological data partition relative to the molecular data influenced the results of the combined analyses. In order to compare and contrast a large number of phylogenetic analyses and their resulting summary trees, we developed a measure for the incongruence between two topologies and simultaneously ignore any differences in phylogenetic resolution. Phylogenetic hypotheses generated using only morphological characters differed among each other, and with previous analyses, whereas molecular-only and combined Bayesian analyses produced extremely similar topologies. Characters historically associated with traditional classification in the Rhynchonellida have very low consistency indices on the topology preferred by the combined Bayesian analyses. Overall, this casts doubt on the use of morphological systematics to resolve relationships among the crown rhynchonellide brachiopods. However, expanding our dataset to a larger number of extinct taxa with intermediate morphologies is necessary to exclude the possibility that the morphology of extant taxa is not dominated by convergence along long branches.

Modern systematics is predominantly founded on the concept of inferring relationships from character data, which may consist of phenotypic (e.g., morphological) characters but, today, is more often performed using character data from molecular sequences. Molecular data are often considered to be less ambiguous, more easily reproducible, and less susceptible to convergence relative to morphological data and thus presumed to possess a higher signal-to-noise ratio (Scotland et al. 2003; Gaubert et al. 2005). Lengthy training is often needed to gain the necessary expertise in the anatomy of a given group of organisms before morphological data can be collected (Wiens 2004). For these reasons, when disagreements arise between phylogenetic analyses based on morphological and molecular datasets, phylogenies based on molecular data are sometimes given preference (Scotland et al. 2003).

However, paleontologists are almost entirely limited to using the morphology of fossil organisms to place fossil taxa in the tree of life. The integration of paleobiology with evolutionary biology via phylogenetics holds considerable promise for understanding macroevolution (Slater and Harmon 2013; Hunt and Slater 2016), and thus it is paramount that we comprehend how to best use morphology for inferring relationships (Guillerme and Cooper 2016; Wright et al. 2016). We must evaluate how much trust we can place in morphological data to reliably reveal such relationships. This is particularly important when morphological and molecular datasets disagree on inferred relationships among living taxa. Shared morphology likely does reflect characters gained via shared ancestry (i.e., homology) in some groups, although in other groups, shared morphology may more closely reflect ecological and other extrinsic constraints on biological form. Determining which of these patterns applies in any specific case requires untangling the developmental, ecological, and evolutionary context of each group (Losos et al. 2012; Zou and Zhang 2016).

We must not get lulled into a false sense of security when we are limited to relying solely on morphological data in paleontology, such as with groups that have rich fossil records but very few or no members surviving to today. We often see disagreements between molecular and morphological datasets in groups with extant members, and thus we should expect the same even when molecular data aren’t available. Conflicts between inferences based on different datasets suggest problems with at least one of the datasets. Morphological data may be problematic due to mistakes in attributing homology, recognizing too few characters or the oversplitting of characters and states (Brazeau 2011), but molecular data could have issues due to a poor alignment, sequence misidentification, or simply that the sampled region for sequences are too quickly evolving to be useful for phylogenetic analysis. Of course, both molecular and morphological analyses can suffer from issues that can impact any phylogenetic such as poor outgroup selection or long-branch attraction (Felsenstein 1978) among selected taxa. Both can also be impacted by problematic methods decisions, such as choosing inappropriate models or priors in a Bayesian analysis, and morphological analyses likely suffer more from this, due to simply having experienced less development of methods and models.

If we wish to determine whether there are specific issues related to morphological datasets, we must assess the reliability of morphological character data. One route to assessing the reliability of morphological character data would be to compare it to an independent molecular dataset, for some group that contains a number of living species, and investigate the source of discrepancies between the two datasets.

Extant brachiopods of the Order Rhynchonellida (Kuhn 1949) are one such group, where previous morphological (Schreiber et al. 2013) and molecular datasets (Cohen and Bitner 2013a) exist but suggest very different patterns of relationship (Fig. 1). Even before these studies were completed, uncertainty in the relationships within Rhynchonellida was illustrated by the fact that it is the only order of rhynchonelliform brachiopods in which superfamilies had not been united into suborders (Manceñido et al. 2007; Savage 2007). Rhynchonellide brachiopods first appear stratigraphically in the Middle Ordovician (Darriwilian; 467–458 Ma) and thus are the earliest and most basal of the extant rhynchonelliform orders (Williams et al. 1996; Savage et al. 2002; Carlson 2007, 2016). Rhynchonellida is morphologically distinct from other brachiopod orders: typically biconvex, astrophic, impunctate, and uniplicate, commonly with costae forming a characteristic zigzag commissure and with well-developed deltidial plates, dorsal median septum, hinge plates, and calcareous lophophore support structures known as crura, which support the base of the lophophore surrounding the mouth (Savage 1996; Savage et al. 2002; Carlson 2016). Rhynchonellida is thought to be a paraphyletic group, sharing common ancestry with some (but not all) syntrophiidine (Ulrich and Cooper 1936) pentameride brachiopods and giving rise to several extinct orders: the Atrypida, Athyridida, Terebratulida, and, possibly, the Spiriferida, Spiriferinida, and Thecideida. (Carlson 1993; Carlson et al. 2002). Rhynchonellida is comprised of over 500 extinct genera, represented today by only 19 extant genera (Savage et al. 2002; Manceñido et al. 2007; Savage 2007). Fewer than 5% of Rhynchonellide genera are extant, thus straining our confidence in placing the vast number of extinct lineages based on morphological phylogenetics alone, in the absence of molecular data, and makes the discordance between analyses of extant taxa all the more troubling.

**Figure 1:**
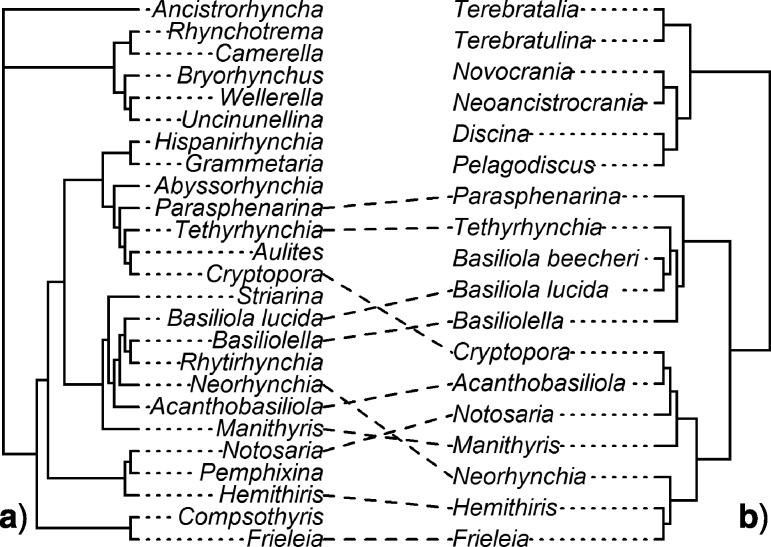
Comparison of published morphological a) and molecular b) phylogenies for the Rhynchonellida. One way to display disagreement between two phylogenetic topologies is a graphical “tangle-gram,” where nodes are optimally rotated so that matches between tip taxon labels in topologies positioned horizontally are maximized. Lines are drawn between identical taxon pairs, such that crossing lines now indicate incongruent relationships. The tree on the left a) is the majority-rule consensus from Schreiber et al. (2013)’s morphological analysis under maximum parsimony, with characters reweighted relative to their consistency indices (as depicted in their fig. 4). The phylogeny on the right b) is the half-compatibility Bayesian posterior phylogeny from Cohen and Bitner (2013a, their Fig. 3), based on SSU and LSU rDNA. This “tangle-gram” figure was constructed using the R library “phytools,” v0.5-20 (Revell 2012). Relationships have been collapsed to the generic level. Superfamilies are not labeled on these phylogenies, due to the high incongruence of those higher taxa with any existing phylogenetic hypothesis (Cohen and Bitner 2013a; Schreiber et al. 2013).

The contrast between the phylogenies preferred by the Schreiber et al. (2013) and Cohen and Bitner (2013a) analyses can be depicted as a “tangle-gram” (Fig. 1), the consideration of which suggests that several factors unrelated to the character data may have exaggerated the apparent discordance. A large number of taxa are unshared between the two analyses, particularly out-group taxa: several non-rhynchonellide taxa were used by Cohen and Bitner, and a number of fossil rhynchonellide taxa used as uncertain outgroups by Schreiber et al. Furthermore, the molecular data were only available for a portion of the extant rhynchonellide diversity, in contrast to the morphological analyses. These differences may have played a large role in the apparent discordance between the analyses.

In this study, we attempt to determine the source of the conflict by reanalyzing the morphological data and combining the morphological and molecular datasets into a single analysis (sometimes also referred to as a “total evidence” analysis; Eernisse and Kluge 1993). First, we reformulate the morphological dataset from Schreiber et al. to increase the comparability of the two datasets by coding the non-rhynchonellide out-groups used by Cohen and Bitner. We then infer phylogenies from a combined dataset of molecular and morphological characters, using both maximum parsimony and partitioned model-based Bayesian analysis (Nylander et al. 2004), under a range of taxa sampling schemes. Although maximum parsimony analysis is the most commonly used approach for morphological data, Bayesian inference may partially avoid issues related to differences in the size of morphological and molecular datasets, as the topologies favored are no longer directly linked to the number of characters but rather the likelihood of observing the character data, given a tree with branch lengths. Thus, if the molecular data are homoplastic due to a relatively fast rate of sequence evolution, and the morphological data appear to be a better indicator of homology, we would expect the morphological data to exert more influence under Bayesian criteria than under maximum parsimony criteria. However, as the number of characters might still impose an effect, we develop and apply a novel test to evaluate the degree to which a morphological dataset of a given size, with very strong phylogenetic signal, could impact a combined analysis.

## Methods

### Initial Datasets

Molecular data were taken from Cohen and Bitner’s (2013a) published dataset of 3435 ribosomal DNAbase pairs (365 parsimony-informative base pairs) for 18 species, 12 of which are rhynchonellide species (and containing 11 of the 19 extant rhynchonellide genera). This dataset is available from their data repository (Cohen and Bitner, 2013b; doi: http://dx.doi.org/10.5061/dryad.79411) and GenBank accession numbers are listed in Supplementary Appendix S3 available on Dryad at http://dx.doi.org/10.5061/dryad.31048.2. The initial morphological dataset was a 58-character matrix for 25 genera (including all 19 extant rhynchonellide genera) from Schreiber et al. (2013), obtained directly from the authors. Schreiber et al. did not sample from outside Rhynchonellida, choosing to use five extinct Paleozoic rhynchonellide brachiopods and one pentameride as out-groups. Ideally, selected out-groups would be as closely related to the in-group as possible to best inform character polarity, but there is no strong evidence that these extinct taxa are clearly outside the crown Rhynchonellida and thus are suboptimal out-group choices. In addition, preliminary analyses with these fossil taxa found that their precise position was very uncertain, resulting in poorly resolved topologies and weak convergence of attempted Bayesian analyses. Based on this behavior, we removed these six taxa from our datasets for all analyses presented here.

Our combined dataset included 20 rhynchonellide taxa along with an additional six non-rhynchonellide taxa included in Cohen and Bitner, for a total of 26 total taxa. We chose to root our analyses in this study at the split between the four inarticulate out-group taxa (*Novocrania*, *Neoancistrocrania*, *Pelagodiscus*, and *Discinisca*) and the remaining articulate taxa, composed of both the rhynchonellide taxa and the two terebratulide outgroup taxa (*Terebratulina* and *Terebratalia*). This split was always obtained in molecular, morphological, and combined analyses. This matches the routine used by Cohen and Bitner (2013a).

### Expanded Morphological Character Coverage and Matching to Molecular Data

As none of the non-rhynchonellide out-group taxa from Cohen and Bitner were coded for morphological characters by Schreiber et al., we expanded the morphological matrix from Schreiber et al. to encompass the inarticulate and terebratulide out-groups for our analyses. Of the 26 taxa, 18 included base pair data from Cohen and Bitner 2013a. Sixty-six interior and exterior shell characters in 147 states were delineated (Supplementary Appendix S1 available on Dryad; Savage 1996, 2007; Savage et al. 2002; Manceñido et al. 2007). The majority of characters are associated with the cardinalia (e.g., presence or absence of a cardinal process), defined as structures of the postero-median portion of the dorsal valve, including crura. External shell characteristics include shell shape, commissural shape, shape of deltidial plates, and shape of foramen. Internal features include shape of dental plates and the size of the hinge plates, cardinal process, median septum, and muscle scars. Crural length, cross-sectional shape, and distal end shape were also characterized (Savage et al. 2002; Manceñido et al. 2007; Schreiber and Carlson 2009). Eight new characters were added to the 58 characters coded by Schreiber et al. (2013); with the addition of inarticulated and terebratulide brachiopods, a greater number of characters varied among the included taxa (e.g., lophophore type). For parsimony-based analyses, characters were defined as either binary or multistate and treated as unordered, fully reversible, and equally weighted (typical Bayesian analyses also assume that morphological characters are unordered and fully reversible). A character was coded as missing for a genus if the character state is unknown or inapplicable for the representative species. Although originally only 19 of these taxa were included in the morphological dataset from Schreiber et al., all 26 taxa were included in our revised morphological matrix (Supplementary Appendix S2 available on Dryad).

Morphological data were coded at the genus level, primarily based on the type species of each genus, as type species generally are the best described for the purposes of morphological description. The only exception to this was the genus *Basiliola* (*Basiliola beecheri* is the type species, but *Basiliola lucida* was used for the basis of determining morphological characters). However, many of the species included in Cohen and Bitner’s analysis are not the type species of their particular genus. We made the simplifying assumption that the morphological character data did not vary within genera, which agrees with our own expert opinion of this group, and for the characters we used. This assumption allowed us to match the species-level molecular data on a one-to-one basis to the genus-level morphological data, by lowering the taxonomic resolution to the genus level. For the single genus (*Basiliola*) sampled for multiple species in Cohen and Bitner (2013a), we appended identical genus-level morphological data to both congenerics (*B. beecheri* and *B. lucida*). Thus, although these two species are included in our combined analyses as having identical morphological data, they have nonidentical sequence data. For the combined Bayesian analyses, this should help inform the relative difference in rates of evolution between the morphological and molecular partitions.

We performed a series of Bayesian and maximum parsimony phylogenetic inference analyses to identify conflicting phylogenetic signals present in molecular and morphological characters among the extant rhynchonellide brachiopods (labels for each analysis are given in Table 1), which varied in taxonomic composition, model settings, and the evaluated character data. Analyses were run on two taxonomic selections, to control for the presence of missing character data (Table 1). Although the final matrix has 66 characters for all 26 taxa (the “all taxa” dataset), only 18 taxa have molecular sequence data from Cohen and Bitner. Restricting to those taxa with both molecular and morphological data, this subset is referred to as the “shared” dataset. Of those 66 morphological characters, seven are not parsimony informative and thus were excluded for our analyses here (including for our Bayesian analyses, see details of the Markov model used below), bringing the final total number of morphological characters to 59 for the full dataset. For the shared dataset with only 18 taxa, an additional five characters were no longer parsimony informative and thus were excluded, further reducing the number of characters considered to 54. In addition to varying the taxa included, two Bayesian analyses (Mol-B-18t and Morph-BMaxI-18t), analyzing only the 18 shared across both datasets, were additionally restricted to characters of a single type to identify the baseline morphological-only or molecular-only preferred topologies, which may shift due to less complete taxonomic sampling relative to the original analyses. A further pair of maximum parsimony analyses (Morph-Pars-26t and Morph-Pars-18t) with the morphological data only were performed using the “all” 26 taxa and the “shared” 18 taxa selections.

**Table 1.** Taxonomic, character, and analytical differences among phylogenetic analyses performed in this study

Analysis name	Taxa used	Characters used	Analysis type	Morphological model settings and priors
Morph-Pars-26t	All taxa except out-groups from Schreiber et al.	Parsimony-informative morphological characters only	Parsimony (PAUP)	—
Morph-Pars-18t	All shared taxa	Parsimony-informative morphological characters only	Parsimony (PAUP)	—
Morph-BMaxI-18t	All Shared Taxa	Parsimony Informative Morphological Characters Only	Bayesian (MrBayes)	Maximize Information
Mol-B-18t	All shared taxa	Molecular data only	Bayesian (MrBayes)	—
Comb-Pars-26t	All taxa except out-groups from Schreiber et al.	Molecular data and parsimony- informative morphological characters	Parsimony (PAUP)	—
Comb-Pars-18t	All shared taxa	Molecular data and parsimony- informative morphological characters	Parsimony (PAUP)	—
Comb-BMinA-26t	All taxa except out-groups from Schreiber et al.	Molecular data and parsimony- informative morphological characters	Bayesian (MrBayes)	Minimize Assumptions
Comb-BMinA-18t	All shared taxa	Molecular data and parsimony- informative morphological characters	Bayesian (MrBayes)	Minimize Assumptions
Comb-BMaxI-26t	All taxa except out-groups from Schreiber et al.	Molecular data and parsimony- informative morphological characters	Bayesian (MrBayes)	Maximize Information
Comb-BMaxI-18t	All shared taxa	Molecular data and parsimony- informative morphological characters	Bayesian (MrBayes)	Maximize Information
Simulation	All shared taxa	Molecular data and parsimony- informative binary characters	Bayesian (MrBayes)	Maximize Information

### Details of Phylogenetic Inference Analyses

As described in Table 1, analyses were conducted under both maximum parsimony optimization, using the software PAUP* 4.0b10 (Swofford 2000), and with Bayesian phylogenetic inference, using the software MrBayes v3.2.5 (Huelsenbeck and Ronquist 2001). Bayesian analyses used settings similar to those used by Cohen and Bitner, applying a six-substitution rate model with gamma-distributed rates, but no analysis applied any type of clock model for character changes, as our main interest is in establishing the topology and not dating divergences. Morphological characters were modeled in Bayesian analyses under the Markov model (“Mk”; Lewis 2001), modified to account for the ascertainment bias of only observing parsimony-informative characters (sometimes referred to as “Mk-parsinf”; Allman et al. 2010). Morphological data are generally missing invariant characters (as these are difficult or impossible to enumerate) and are often missing autapomorphic data (for practical reasons, as it is impossible to enumerate the number of static morphological characters for a group). As the data are thus filtered, it is necessary to apply a modified Markov model to properly calculate the likelihood. Although our raw data matrix contains some autapomorphies, their inclusion was not intentional but rather a by-product of defining characters without respect to a particular set of taxa, and the number of invariant and autapomorphic morphological characters increases when the shared taxon subset is considered.

Our morphological analyses with MrBayes were performed under two sets of conditions (Table 2). Unlike the typical approach to maximum parsimony analysis of morphological data, Bayesian phylogenetics infers an explicit model that carries specific assumptions about the process of character evolution, assumptions that can be relaxed a priori. Given the relative uncertainty in what the full effect of relaxing such constraints on morphological analyses might have on the resulting topologies, as Bayesian morphological analyses are still in their relative infancy, we chose to analyze morphological data under two models, one very simple and highly constrained, and the second, very complex and generalized, relaxing as many a priori assumptions as possible. The first of these end-member models (referred to as “Maximum Information” or “MaxI”) attempts to maximize the apparent information content of morphology for phylogenetic inference maximized, under the assumption that morphological evolution could be as simple as supposed by such a constrained model, and thus that fitting a more generalized model may increase uncertainty in parameter estimates, including topological uncertainty (Nylander et al. 2004). While maximizing the potential information content of the data could seem to be synonymous with “increasing accuracy” of our analyses, this approach is biased toward misinterpreting noise in the dataset as meaningful signal, which more relaxed models might properly recognize as meaningless with respect to relationships. The second end-member model minimizes those a priori assumptions, at the cost of decreased phylogenetic certainty from morphological data (“Minimum assumptions” or “MinA”).

**Table 2.** Model configurations used for analyzing morphological data in Bayesian phylogenetics analyses

Morphological model settings and priors	Rate distribution across characters	Branch lengths across partitions	Symmetric Dirichlet hyper-prior on state frequencies
Maximize Information	Equal	Linked	Fixed (infinity)
Minimize Assumptions	Gamma-distributed variation	Unlinked	Uniform (1, 10)

There are three major differences between these two sets, in terms of model settings and choice of priors in MrBayes (Table 2). The first is whether rates of change are assumed to be equal across all morphological characters (the default setting in MrBayes) or whether a Gamma distribution is used to model variation in rates of change across characters. The second is whether branch lengths (i.e., the estimated amount of evolutionary change on branches) are constrained to be the same between the molecular and morphological partitions. If constrained, this setting assumes that similar amounts of morphological and molecular change are expected along each internode branch. If branch lengths are independently inferred for each partition on a shared topology, then the assumption of similar relative change between the partitions is relaxed, removing the ability for information about change from one partition to inform evolutionary change inferred in the other partition.

The third and final difference was the treatment of the symmetric Dirichlet hyper-prior on state frequencies (Wright et al. 2016). This hyper-prior essentially controls the evenness of the transition rates between states (i.e., forward transition) and the reversal transitions (transitions back to the original state). Dirichlet hyper-priors thus reflect our assumption about whether we expect states to be in equilibrium on long time scales, suggesting reversal is as likely as forward transition or whether we expect lineages to convert to particular states over time, because reversal is unlikely. By default, MrBayes assigns a value fixed on infinity, which assumes reversal is exactly as likely as forward transition and thus states are *strongly* expected to occur in equal frequency at equilibrium. As we may have little reason to expect that rates of transition are exactly equal, we can relax this assumption by assigning a smaller value to this hyper-prior, essentially setting a less informative prior on the evenness of state frequencies at equilibrium. Unfortunately, our experience with MrBayes analyses with the symmetric Dirichlet hyper-prior set to values below 1 or with values that can fall below 1 (e.g., exponential distribution with lambda$=1$) appear to consistently interact with the Gamma distribution on rate variation across characters to produce a divide-by-zero error. Trial analyses without Gamma-distributed rate variation, but with a sufficiently flat hyper-prior, generally found mean posterior values for the symmetric Dirichlet hyper-prior between 1 and 3. Thus, a uniform distribution with range (1, 10) was assigned to this hyper-prior for the “Minimize Assumptions” analyses.

Bayesian analyses were performed for 200,000 generations with two independent runs, with each run composed of four chains. This arrangement was found to be more than sufficient to ensure parameter and topological convergence of the independent runs in preliminary analyses. Convergence was checked using inspection of Potential Scale Reduction Factors (PRSF) values (Gelman and Rubin 1992) as reported by MrBayes for each parameter and investigation of the resulting parameter values with Tracer v1.6 (Rambaut and Drummond 2013). Almost all of our final Bayesian analyses presented in this study converged successfully, with standard deviations of splits reported well below 0.01, high PRSF values, effect sample size (ESS) for parameter estimates greater than 1000, and smooth posterior parameter distributions as visualized with Tracer. The one exception was analysis Comb-BMinA-26t, which was continued until stopping at 42,010,000 generations, with an average standard deviation of split frequencies of 0.011. However, PRSF values of parameter values were all effective at 1.00, and the lowest ESS was more than 4000. Visual inspection in Tracer revealed no oddities, other than an irregular posterior distribution for the relative rate parameter for the morphological character partition, with an anomalous peak at 1.00 (for both MCMCMC runs, this parameter’s ESS was more than 19,000).

### Sensitivity Analyses

Two final analyses were performed to assess the degree to which 54 morphological characters could impact an analysis that also includes 3435 molecular characters. The first analysis repeated the original morphological matrix 53 times, to create an artificial morphological partition roughly the same size as the molecular partition (a morphological partition with 2862 characters). This is analogous to the practice of giving different weights to specific characters in maximum parsimony analyses, at least when characters are only weighted with positive integers (and thus count the same as repeating those characters). This dataset failed to converge, however, under a series of combinations of model settings and priors in MrBayes.

**Figure 2:**
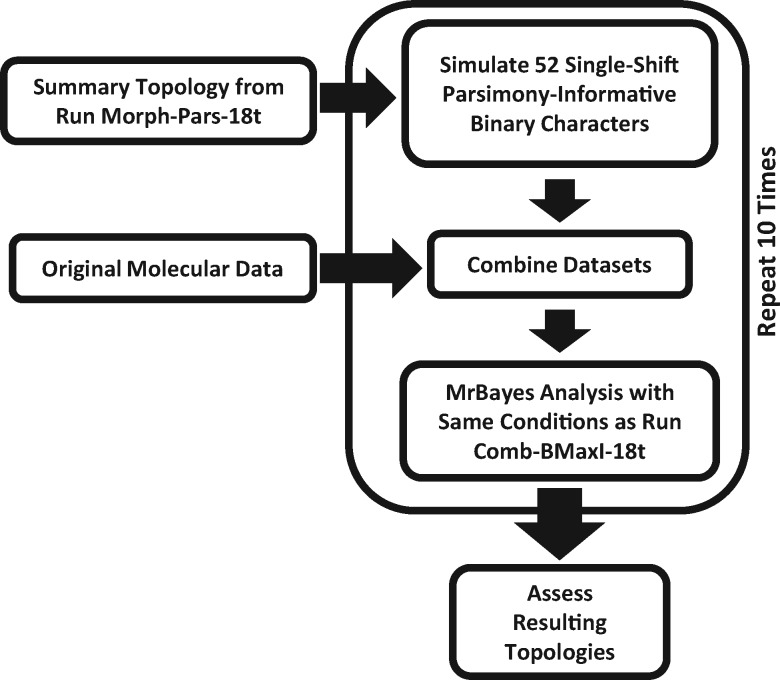
Flowchart illustrating the workflow for the simulation-based sensitivity analyses. In the first step, a combined dataset composed of the original molecular data and a simulated morphological matrix is generated, using the majority-rule consensus summary from analysis Morph-Pars-18t as the basis for simulating the morphological data. This artificial dataset is then analyzed in MrBayes, using the same settings as analysis Comb-BMaxI-18t. The entire process is then replicated 10 times, and the output is summarized and compared with the summary topologies obtained from the empirical analyses.

The second simulation-based sensitivity analysis (Fig. 2) simulated 54 binary, parsimony-informative characters that perfectly support a given morphology-based topology, and we combined these with the existing molecular data. Such simulated morphological characters can be generated without the use of any Markov process, or other stochastic model, by placing single-shift binary characters on the nodes of a selected, unrooted tree topology. This procedure was performed using the function “perfectParsCharTree” in the freely available R package paleotree, v2.7 (Bapst 2013). This approach thus simulates a combined dataset, where a parsimony-informative morphological dataset exists that lacks any homoplasy, and strongly supports a topology preferred by the morphological, is combined with the original molecular data. This removes the confounding effect of conflicting signal in the morphological data and allows us to assess directly whether the size of the morphological dataset is sufficient for impacting the results of a combined analysis. For this analysis, we selected the morphology-only, majority rule consensus produced by PAUP in analysis Morph-Pars-26t, which contains only 13 nodes (ignoring the root). We generated character data by evenly placing changes in 52 of our 54 simulated characters across these 13 nodes. The remaining two such characters could not be placed evenly across 13 nodes, and thus their placement was randomly assigned, and this was repeated 10 times to ensure that their placement did not impact the resulting topology. These data were then combined with the molecular dataset and analyzed in MrBayes with identical model settings as analysis Comb-BMaxI-18t (Fig. 2). These 10 replicates took about 6 days to compute, and produced identical topologies, and thus we judged 10 replicates as sufficient for our purposes.

### A Measurement of Topological Contradiction

Such a large number of separate phylogenetic analyses, with nonidentical sets of taxa across these analyses, present difficulties in assessing the relative degree to which analyses agreed or disagreed with one another. We used summary topologies to simplify the most parsimonious trees and posterior tree samples output by our analyses and make them comparable, although retaining information on topological uncertainty. We summarized our Bayesian and maximum parsimony analyses with half-compatibility trees and majority-rule consensus trees respectively, which are philosophically similar in that they reflect the nodes supported by at least half the Bayesian posterior tree sample or sample of most parsimonious trees.

**Figure 3:**
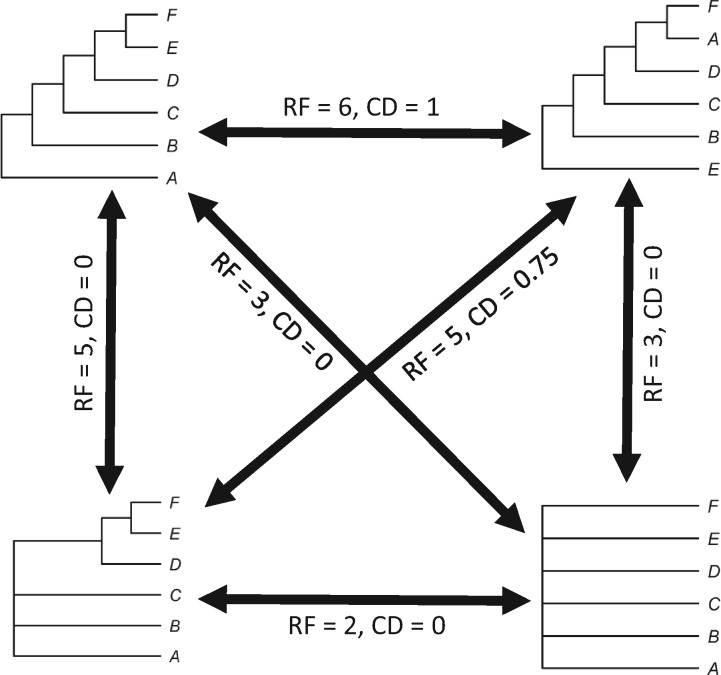
Conceptual diagram of pairwise tree comparisons, illustrating how the typically used Robinson–Foulds metric (RF; Robinson and Foulds 1981) contrasts with the CD measure introduced in this study. Notably, RF penalizes trees for differences due to lack of resolution, but CD simultaneously ignores differences due to lack of resolution, making it ideal for comparing summary topologies. Note that, unlike RF, CD values are scaled to be between 0 and 1.

However, it is not simple to quantify the disagreement between two summary trees, especially when both of topologies may not be fully bifurcating (i.e., not fully resolved). Ultimately, we are interested in whether two summary trees actively contradict each other, not whether one tree has less resolution than the other. Typical pairwise topology metrics, like the classic Robinson-Foulds distance (Robinson and Foulds 1981), are not useful for such comparisons (Fig. 3). For example, nonzero Robinson–Foulds distance exists between a poorly resolved tree and a well-resolved tree even when there are no conflicting relationships; that distance is entirely a function of the number of missing nodes in the less well-resolved tree. Instead, we developed our own topology-comparison measurement, calculated as the total number of conflicting splits across two topologies, after dropping any tip taxa not shared between the two trees. By dividing this number by the maximum number of splits that could conflict between two topologies, $2\times$(number of shared tips $-\,2$), we can scale this value between 0 (i.e., no conflicting relationships) and 1 (two entirely conflicting topologies), converting the measure to a symmetric pairwise distance. Algorithmically, we identified conflicting splits by counting the number of splits on one tree that disagreed with at least one split on the other tree: that is, the taxa segregated by that split were found to be more closely related to a nonoverlapping, different set of taxa, not segregated together via the same split. Thus, the calculation for this “contradiction difference” (CD) value, for two topologies labeled A and B, and following the removal of any tips not shared by both from both topologies, is given as:

$N_{\text{AB}} =$ number of splits on tree A contradicted by one or more splits on tree B
$N_{\text{BA}} =$ number of splits on tree B contradicted by one or more splits on tree A

$$\text{Contradiction difference}=\frac{N_{AB}\;+\;N_{AB}}{2\;(number\;of\;shared\;tips\;-\;2)}.$$

This measure is symmetric, like the pairwise Robinson–Foulds distances, and which tree is labeled A or B is arbitrary. This measure has some unusual, but intentional, mathematical properties. In particular, the CD is not a Euclidean “metric,” as it violates the triangle inequality property. For example, a number of very different well-resolved trees would all be 0 CD from a star tree of the same taxa, although simultaneously having nonzero distances between each other (Fig. 3). However, as we are using this CD value solely as an indicator of the extent of disagreement, such properties are of little concern. Note that this measure, if only applied between fully resolved phylogenies, is essentially a symmetric pairwise Robinson–Foulds distance rescaled to the number of taxa shared across both topologies. Thus, our CD measure is only relevant to analyses comparing among summary topologies, as these are typically the only sort of topologies that might contain polytomies, as the CD ignores differences in phylogenetic resolution. We calculated our pairwise CD measurements between the summary topologies obtained as described above.

All data, input files and programming scripts for recreating all analyses and analytical figures in this study can be found at the Supplementary Material available on the Dryad Digital Repository (http://dx.doi.org/10.5061/dryad.31048.2), with a README file detailing the contents. A supplemental PDF containing figures of all summary topologies considered, with their support values, can be found in our Dryad repository.

## Results

### Morphology-Only Phylogenetic Analyses

**Figure 4:**
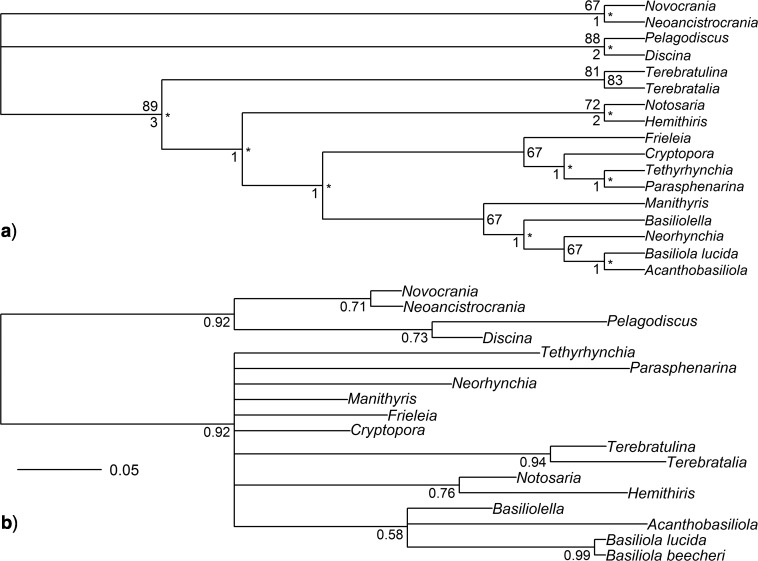
Phylogenies inferred from the revised morphological data only, including only those taxa for which molecular data are also available (Cohen and Bitner 2013a). a) Majority-rule consensus from a maximum parsimony analysis (analysis Morph-Pars-18t). Upper left node labels are bootstrap percentages; values not shown for nodes with bootstraps less than 50%. Center right node labels are the percentage of most parsimonious trees a clade was observed in, with nodes observed 100% indicated by an asterisk (*). Lower left node labels are Bremer support values for nodes with Bremer support values greater than zero. b) Half-compatibility tree from a Bayesian analysis (analysis Morph-BMaxI-18t) with nodes labeled by their posterior probabilities, rounded to two significant digits. Probabilities are not shown when effectively equal to 1.00.

Parsimony analyses based only on the revised morphological dataset (analyses Morph-Pars-26t and Morph-Pars-18t) produced summary trees, both of which contradicted the preferred topology from Schreiber et al. (Fig. 1a), from 0.44 CD for Morph-Pars-26t to 0.67 CD for Morph-Pars-18t (Table 3). Unlike Schreiber et al., who showed the maximum amount of contradictory splits (i.e., 1 CD) with the original molecular topology (from Cohen and Bitner; Fig. 1b), these new morphological trees contained fewer contradictory relationships relative to the molecular topology (0.11–0.56 CD) but that may partly be attributable to including the inarticulate out-groups in the revised morphological data. Interestingly, the Bayesian analysis of the revised morphological data (analysis Morph-BMaxI-18t) resulted in a half-compatibility tree that was relatively poorly resolved, and the topology contradicted little with the PAUP analyses (Morph-Pars-26t and Morph-Pars-18t; 0–0.07 CD) but had relatively good agreement with the original Schreiber et al. topology (0.11 CD). This suggests that analysis Morph-BMaxI-18t’s half-compatibility topology (Fig. 4b) may be poorly resolved but could be reasonably described as a “compromise” topology for the morphology-only analyses, with those areas that are well resolved being fairly consistent across the different morphological analyses. Any topology that disagrees strongly with this “compromise” would also disagree strongly with the entire set of morphology-only analyses, including those that are better resolved than the “compromise.”

**Table 3.** Pairwise CD measurements between two topologies taken from previous studies, and the summary topologies from the phylogenetic analyses performed in this study

	Schreiber et al.	Morph-Pars-26t	Morph-Pars-18t	Morph-BMaxI-18t	Mol-B-18t	Comb-Pars-26t	Comb-Pars-18t	Comb-BMinA-26t	Comb-BMinA-18t	Comb-BMaxI-26t	Comb-BMaxI-18t	Simulation (a)
Cohen and Bitner	**1**	0.67	0.67	0.41	0.16	0.53	0.31	0.19	0.19	0.16	0.22	0.50
Schreiber et al.		0.41	0.56	0.11	0.89	0.72	0.83	0.61	0.83	0.83	0.89	0.78
Morph-Pars-26t			0.33	**0**	0.53	0.50	0.57	0.40	0.50	0.50	0.53	0.47
Morph-Pars-18t				0.07	0.57	0.63	0.63	0.47	0.57	0.53	0.57	0.60
Morph-BMaxI-18t					0.31	0.31	0.34	0.19	0.28	0.28	0.31	0.28
Mol-B-18t						0.34	0.12	**0**	**0**	**0**	0.06	0.31
Comb-Pars-26t							0.19	0.38	0.28	0.42	0.28	0.06
Comb-Pars-18t								0.12	0.12	0.12	0.12	0.31
Comb-BMinA-26t									**0**	**0**	**0**	0.22
Comb-BMinA-18t										**0**	**0**	0.25
Comb-BMaxI-26t											**0**	0.25
Comb-BMaxI-18t												0.25

Analyses are labeled as listed in Table 1. As the CD is symmetric, only the upper triangle of this table is shown. Values of CD at 0 or 1 are in boldface.

### Bayesian Molecular-Only and Combined Dataset Analyses

**Figure 5:**
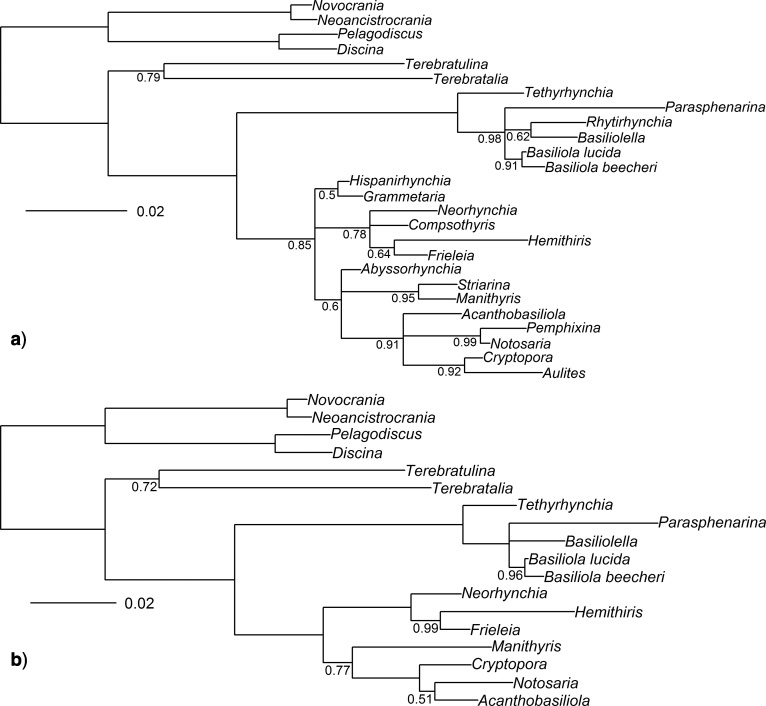
Half-compatibility summaries from Bayesian analyses appliedto a combined dataset of the molecular and revised morphologicaldata, under strictest model and prior settings for the morphologicalcharacters. a) Analysis Comb-BMaxI-26t, containing all taxa in thedataset including extant taxa with no molecular data. Rhynchonellidegenera are labeled by their current superfamily membership(Savage et al. 2002): respectively, Dimerelloidea (D), andHemithiridoidea, (H) Norelloidea (N), and Pugnacoidea (P). b)Analysis Comb-BMaxI-18t, containing only those taxa that have bothknown morphological and molecular data. Nodes are labeled with theirposterior probabilities, rounded to two significant digits.Probabilities are not shown when effectively equal to1.00.

Combined data analyses with MrBayes (i.e., analyses Comb-BMaxI-26t, Comb-BMinA-26t, Comb-BMaxI-18t, and Comb-BMinA-18t; Fig. 5) were nearly always congruent with each other (i.e., CD at or close to 0). For the purposes of choosing a single analysis to compare with others, we chose analysis Comb-BMaxI-18t, the Bayesian combined analysis of only those taxa with both morphological and molecular data, using strict model assumptions. Analysis Comb-BMaxI-18t’s half-compatibility topology (Fig. 5b) has no incongruent relations with analyses Comb-BMaxI-26t, Comb-BMinA-26t, or Comb-BMinA-18t (0 CD). In general, the Bayesian combined data analyses have little to no disagreement with our Bayesian reanalysis of the molecular data (Mol-B-18t), with 0 CD between analysis Mol-B-18t, versus analyses Comb-BMaxI-26t, Comb-BMinA-26t, and Comb-BMinA-18t. Analysis Comb-BMaxI-18t versus Mol-B-18t has a CD of 0.06, due to a small shift in whether *Acanthobasiliola* is more closely related to *Notosaria* or *Cryptopora*. However, this difference is barely supported in either analysis, with the posterior probability for *Cryptopora* as sister to *Acanthobasiliola* being 0.55 in analysis Mol-B-18t and the posterior probability for *Notosaria* and *Acanthobasiliola being sisters is* 0.51 in analysis Comb-BMaxI-18t. The original Cohen and Bitner molecular analysis is less congruent with these combined analyses than Mol-B-18t, which agreed with the combined Bayesian analyses in placing *Tethyrhynchia* in a less nested position than in Cohen and Bitner’s analysis. None of the Bayesian combined analyses produce topologies that resemble those produced by any of the morphology-only analyses. Even the poorly resolved “morphology-only compromise” topology resulting from analysis Morph-BMaxI-18t (Fig. 4b) infers relationships within rhynchonellides that are incongruent with Bayesian analyses of combined data, despite that topology leaving many of the taxa in an unresolved polytomy (e.g., the CD between analyses Morph-BMaxI-18t and Comb-BMaxI-18t is 0.31).

### Maximum Parsimony Combined Dataset Analyses

**Figure 6:**
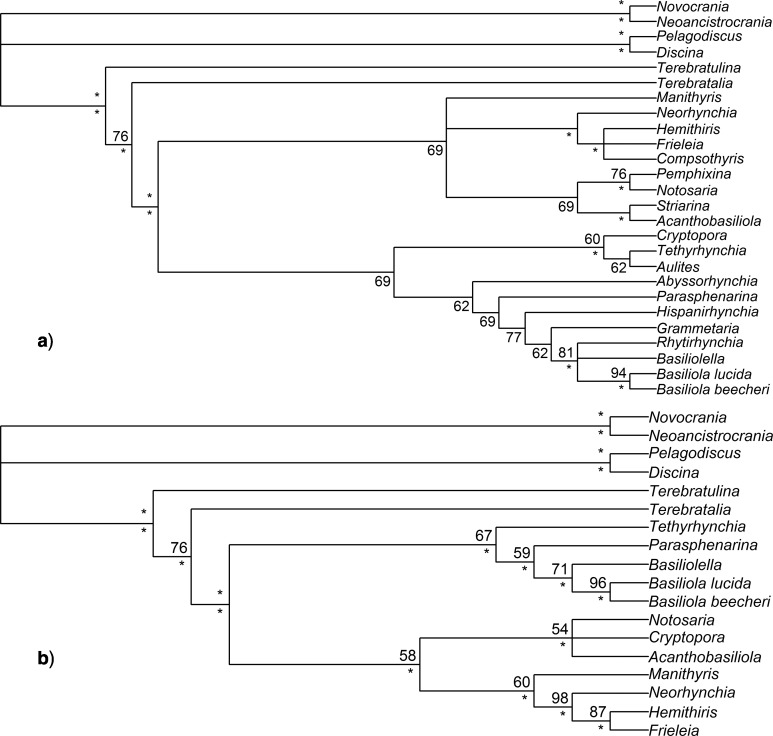
Majority rule consensus trees from maximum parsimony analyses applied to a combined dataset of the molecular and revised morphological data. These are respectively a) Analysis Comb-Pars-26t, with all taxa considered, and b) Analysis Comb-Pars-18t, containing only those taxa that have both known morphological and molecular data. For both cladograms, two node labels are shown. Upper left node labels are bootstrap percentages; values not shown for nodes with bootstraps less than 50% and values at 100% indicated by an asterisk (*). Lower left node labels are the percentage of most parsimonious trees in which a clade was observed, with nodes observed 100% indicated by an asterisk (*).

The all-taxa maximum parsimony combined data analysis (Comb-Pars-26t) disagrees with various Bayesian combined data analyses (0.28–0.42 CD) and disagrees with morphology-only analyses (e.g., 0.31 CD with analysis Morph-BMaxI-18t). Much of this disagreement comes from analysis Comb-Pars-26t moving the majority of taxa without molecular data into a single, pectinate sub-clade, resulting in considerable disagreement with analyses Comb-BMaxI-26t and Comb-BMinA-26t (Fig. 6a). These morphology-only taxa have a substantial effect on the maximum parsimony analyses, as their removal in the shared-taxon PAUP analysis (Comb-Pars-18t) leads to considerable incongruence between the two (0.19 CD). Analysis Comb-Pars-18t also disagrees with the Bayesian combined analyses, although to a lesser extent (0.12 CD with all), due to a shift in the placement of *Manithyris* and due to a shift of the two terebratulides toward paraphyly with respect to the rhynchonellides (Fig. 6b). This latter result of paraphyly was produced only in analyses Comb-Pars-26t and Comb-Pars-18t; the terebratulides are monophyletic in every other analysis. As with analysis Comb-Pars-26t, analysis Comb-Pars-18t is in relatively good agreement with the morphology-only analyses (0.34 CD with analysis Morph-BMaxI-18t).

### Simulation-Based Sensitivity Analyses

**Figure 7:**
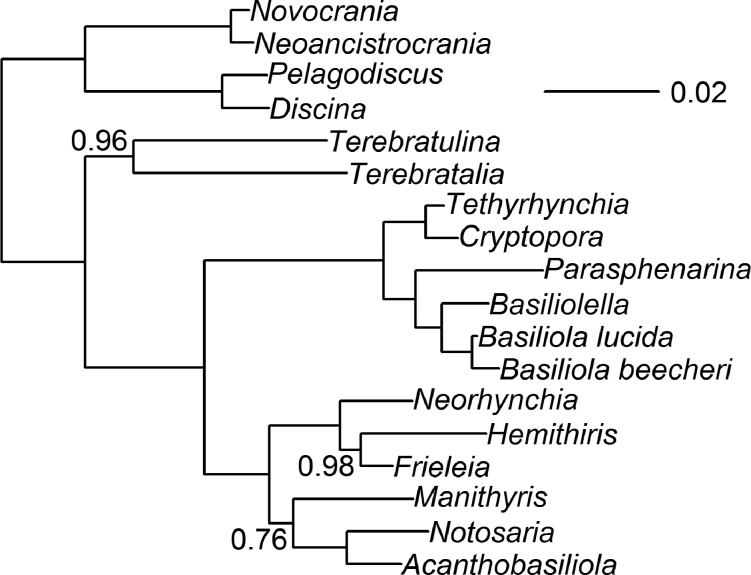
half-compatibility summary from one iteration of the sensitivity analysis, where the molecular data were combined with simulated “perfect” morphological data. The latter is a matrix of binary characters, the same size as our revised matrix, with state transitions evenly placed without homoplasy on nodes of the majority rule consensus topology from analysis Morph-Pars-26t (displayed in Fig. 4a). Nodes are labeled with their posterior probabilities, rounded to two significant digits. Probabilities are not shown when effectively equal to 1.00. All iterations of the sensitivity analyses produced half-compatibility summaries with identical topologies (see Supplementary Material available on Dryad).

To test whether the relative size of data partitions impacted the combined analyses, we performed simulation-based sensitivity analyses, with 10 iterations (Fig. 2). This simulation approach controls for any potential homoplasy or poor signal within the morphological data, so we can test whether 54 morphological characters (the number of parsimony informative morphological characters in the “shared” 18 taxon* Combined Dataset Analyses* dataset) have the potential to impact an analysis when combined with 3435 ribosomal DNA sequence characters. These 10 simulation replicates all produced an identical half-compatibility topology (Fig. 7), similar to a mixture of topological features from analysis Comb-BMaxI-18t (0.25 CD) and Comb-Pars-26t (0.06 CD). Relative to analysis Comb-BMaxI-18t, which may be the better comparison to the sensitivity analyses, given the model settings under which the simulations were performed, the incongruence is due to *Cryptopora* shifting from being sister to *Notosaria* and *Acanthobasiliola* (a relationship seen in all of the molecular and Bayesian combined analyses), and instead to become sister to *Tethyrhynchia*. Relative to analysis Comb-Pars-26t, the only difference with the simulation-based analyses is that *Manithyris* has moved to a slightly more nested position. The simulations disagreed with the “compromise” of the morphology-only simulations, just as the various Bayesian combined data analyses did (e.g., 0.28 CD vs. analysis Morph-BMaxI-18t).

## Discussion

### The Relative Phylogenetic Signals of Morphological and Molecular Data in the Rhynchonellida

The original maximum parsimony morphology-only analyses and the new analyses presented here, based on a revised matrix, produced highly incongruent results, but all were in relative agreement with a poorly resolved output of a Bayesian analysis on the same data. This similarity of the morphology-only analyses with the Bayesian “compromise” is not an artifact of the low resolution of the Bayesian analysis alone, as the morphology-only compromise topology was relatively incongruent with the results of the various combined dataset analyses (Table 3). This pattern agrees with recent simulation-based studies that found that maximum parsimony analyses of morphological datasets are often better resolved than Bayesian analyses but are also typically less accurate than Bayesian analyses (Wright and Hillis 2014; O’Reilly et al. 2016; Puttick et al. 2017). The apparent sensitivity of the morphology-only analyses to minor changes to the taxa included or characters used suggests that the morphological data may have poor phylogenetic signal. Alternatively, the molecular-only analysis (Mol-B-18t) and combined-data Bayesian analyses produced highly congruent results, regardless of changes in model settings and the particular selection of included taxa. That the Bayesian combined topologies and the Bayesian molecular topology rarely contradict one another suggests that these analyses are robust to differences in assumptions, although some analyses did produce somewhat poorly resolved summary trees (e.g., analysis Comb-BMinA-26t, which suffered from convergence issues; see Methods).

The disagreement between the morphology-only analyses and all combined analyses (both Bayesian and maximum parsimony) suggests that, in general, the signal of the molecular data is stronger than that of the morphological data. However, the apparent difference in signal may actually be due to a difference of two orders of magnitude in the size of the partitions, and for this reason, we attempted sensitivity analyses to test this possibility. When we simulated noiseless character data of the same size as our morphological matrix, and combined these data with the molecular data in the sensitivity analyses, we obtained a topology that was nearly as incongruent with the combined analyses (0.25 CD with analysis Comb-BMaxI-18tF) as with the morphology-only analyses (0.28 CD with analysis Morph-BMaxI-18t). Overall, this suggests that even a 54-character morphological matrix can impact a 3435-character molecular partition. Including the morphological data apparently produced one tangible difference in topology between the half-compatibility summaries for combined analysis Comb-BMaxI-18t and molecular analysis Mol-B-18t, but that difference was very poorly supported, based on posterior probabilities.

The results of the maximum parsimony combined analyses (analyses Comb-Pars-26t and Comb-Pars-18t) did not particularly resemble the molecular-only analysis, unlike the Bayesian combined analyses. The close resemblance of the all-taxa, combined-data, PAUP analysis (Comb-Pars-26t) and the sensitivity simulations, simulated with “perfect” morphological signal, could imply that the maximum parsimony combined analyses differ from the Bayesian combined analyses because they allow the morphological data to have more weight in the analysis. However, analyses Comb-Pars-26t and Comb-Pars-18t disagreed with each other and both disagreed even more with morphology-only analysis Morph-BMaxI-18t. This suggests that both may have found two different, alternative regions of tree space that maximize parsimony optimality, but simultaneously differ from the results of the Bayesian analyses. Given their additional discordance with the morphology-only maximum parsimony analyses, perhaps the molecular data are susceptible to factors typically considered responsible for incongruence between parsimony-based and model-based analyses, such as long-branch attraction. Thus, the Bayesian combined analyses in this study, not the maximum parsimony analyses, may be granting the morphological data the greatest impact on resulting topologies. Simultaneously, the molecular data appears to suffer from potential artifacts under parsimony.

### Role of Missing Morphological and Molecular Data

Wiens (2009) suggested that molecular data could play a powerful role in combination with morphological datasets, by indicating which morphological characters had relatively more phylogenetic signal, based on their congruence with the molecular data, and thus help place taxa only known from morphological data, such as extinct taxa. In general, the placement of taxa lacking molecular data in our analyses was uncertain in our combined analyses. Although the half-compatibility tree from combined analysis Comb-BMaxI-26t was relatively well resolved, analysis Comb-BMinA-26t, which relaxed many of the assumptions of Comb-BMaxI-26t, had issues converging (i.e., the independent MCMC runs were not converging on the same space of topological relationships and parameter values), and only a poorly resolved consensus was obtained. The majority-rule topology from the maximum parsimony combined analysis (Comb-Pars-26t) was well resolved, but disagreed considerably with the topology from analysis Comb-BMaxI-26t (0.42 CD).

One likely explanation for the disagreement in the placement of morphology-only taxa concerns the lack of extinct taxa on our analyses. Including morphological “intermediates” from the fossil record might provide combinations of both primitive and derived morphological characters and that subdivide morphological gradients more finely, avoiding potential long-branch attraction from phenotypic convergence between distantly related lineages (Wiens 2009).

The age of the Rhynchonellida crown clade might be very shallow (Cenozoic) or very deep (Paleozoic) depending on the phylogenetic relationships among extinct and extant lineages (Carlson 2016). These relationships have not yet been investigated comprehensively, in part because the number of extinct rhynchonellide taxa vastly outnumbers the extant taxa. Incongruence between the current classification (morphology-based) and applied phylogenetics (based on molecular, morphological, and combined data) of extant taxa only underscores the differences in the two sources of data and makes it difficult to separate the concepts of crown clade from total clade at this time. The morphological data appear to be noisy relative to the molecular data, suggesting high homoplasy in the morphological data. This may imply that it will be difficult to ever assemble a well-supported hypothesis of relationships for both the extant and extinct Rhynchonellida. Alternatively, it may reflect an ancient age of the crown clade, and thus a long period of geological time allowing for substantial morphological evolution to occur. So much character evolution could occur that it would be extremely difficult to reconstruct rhynchonellide relationships based on data from extant taxa alone, with so many morphologically intermediate taxa within the crown clade now extinct, perhaps leading to saturation of the potential combinations of morphological characters (Wagner 2000). Dense taxonomic sampling of the rich rhynchonellide fossil record for phylogenetic analyses is necessary to clarify the question of how old the crown clade is. Including fossils has been observed to improve agreement between discordent molecular and morphological analyses in other groups (e.g., Legg et al. 2013).

Missing data are also an issue for the original sequence data. Cohen and Bitner (2013a) sampled all taxa for small-subunit ribosomal DNA but were unable to obtain samples of large-subunit rDNA for several taxa. Furthermore, some taxa had a large deletion within the short-subunit rDNA. When we evaluated the percentage of missing data in the two partitions (Table 4), we discovered that several taxa lacked more than 50% of the total possible molecular sequence characters: *Acanthobasiliola*, *Cryptopora*, and *Neorhynchia*. The first two are always closely related (if not sister taxa) in the molecular-only and Bayesian combined analyses, usually with *Notosaria* (which is sometimes sister to *Acanthobasiliola*). *Cryptopora* and *Acanthobasiliola* show no such close unity in any of the morphology-only datasets. Incongruence between analysis Comb-Pars-26t and the other Bayesian combined analyses is driven mainly by *Cryptopora* placing in a distant, derived position, far from *Acanthobasiliola*. The shift of *Cryptopora* away from *Acanthobasiliola* is also the cause for the incongruence between the simulations and the combined Bayesian analyses. Overall, this suggests that the close relationship of *Cryptopora* with *Acanthobasiliola*, which appears to be strongly supported based on posterior probabilities (generally higher than 0.9), may actually be a fragile association created by their shared lack of molecular data, easily altered with a small amount of contradictory morphological data. In contrast to the molecular data, the only taxa missing excessive amounts of morphological character data are the inarticulate out-groups because of the few characters they share with Rhynchonellida due to their very distant shared ancestry. The only evidence in conflict with this interpretation is the fact that *Neorhynchia*, the third taxon missing a majority of its (potential) sequence data, typically is placed distantly from both *Acanthobasiliola* and *Cryptopora* in analyses that include molecular data.

**Table 4.** Percentage of missing (or deleted) molecular sequence data and missing morphological character data for each taxon

	Molecular (%)	Morphological (%)
	
*Abyssorhynchia*	—	10.6
*Acanthobasiliola*	**51.3**	12.1
*Aulites*	—	10.6
*Basiliola beecheri*	40.5	10.6
*Basiliola lucida*	46.3	10.6
*Basiliolella*	00.1	16.7
*Compsothyris*	—	04.5
*Cryptopora*	**66.2**	07.6
*Discina*	26.4	**68.2**
*Frieleia*	00.6	06.1
*Grammetaria*	—	06.1
*Hemithiris*	25.6	00.0
*Hispanirhynchia*	—	06.1
*Manithyris*	26.9	04.5
*Neoancistrocrania*	25.6	**77.3**
*Neorhynchia*	**51.2**	06.1
*Notosaria*	00.1	00.0
*Novocrania*	00.1	**72.7**
*Parasphenarina*	25.7	12.1
*Pelagodiscus*	26.3	**71.2**
*Pemphixina*	—	00.0
*Rhytirhynchia*	—	10.6
*Striarina*	—	07.6
*Terebratalia*	00.1	12.1
*Terebratulina*	00.1	16.7
*Tethyrhynchia*	42.1	18.2

Dashes in the first column reflect taxa for which no molecular sequence data are known. Values shown are rounded to the first decimal place. Values over 50% missing are in boldface.

### Morphological Phylogenetics in the Rhynchonellida

Studies that combine morphological and molecular data allow us to evaluate whether the morphological characters traditionally used to define higher-order classifications in a group individually bear any congruence to a second dataset. Although it appears that our morphological data as a whole do not bear a consistent signal across different phylogenetic analyses, it is possible that particular morphological characters may be consistent with the topology preferred by the molecular-only analyses and the combined phylogenies. Furthermore, as both the previous morphological (Schreiber et al. 2013) and molecular analyses (Cohen and Bitner 2013a) found topologies at odd with traditional classifications of the rhynchonellides, traditional systematic characters may not be as informative about evolutionary groupings as previously thought (Savage et al. 2002; Manceñido et al. 2007; Savage 2007). However, this inference does not reject the possibility that other characters may be informative, and it is important to test whether there is any hidden, secondary morphological support for the molecular topology. A recent study found that morphological phylogenetic analyses had better agreement with molecular analyses in mammals, after the morphological dataset was restricted to only those characters with a low level of relative convergence (Zuo and Zhang 2016).

**Figure 8:**
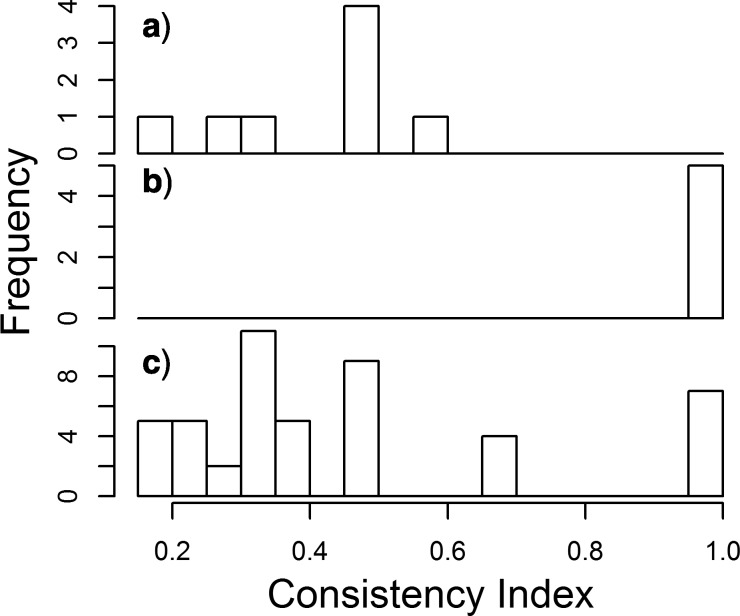
The morphological characters most consistent with the combined analysis are not typically used within the Rhynchonellida. a–c) are histograms of consistency indices calculated using the half-compatibility topology from analysis Comb-BMaxI-18t (combined dataset), for three morphological character subsets. a) Those characters often considered to be useful for systematic purposes in the Rhynchonellida (characters 1, 3, 6, 7, 8, 11, 41, 47). b) Those characters used for distinguishing articulated brachiopod lineages from inarticulated out-groups, which do not vary among the Rhynchonellida (characters 59, 61, 62, 65, 66). c) Remaining characters. The remainder with high consistency (12, 23, 26, 28, 30, 44, 53) have high consistency indices due to rhynchonellide taxa for which that character is unknown or inapplicable.

We measured the consistency indices for our 66 morphological characters on the half-compatibility topology from analysis Comb-BMaxI-18t, our preferred Bayesian combined analysis. The consistency index (Kluge and Farris 1969) varies from 0 to 1 relative to the amount of apparent homoplasy observed in a character, with 0 representing seemingly highly convergent characters and 1 representing characters that appear to have no homoplasy. We found a bimodal distribution consisting of characters with both strong and poor consistency (i.e., low and high homoplasy, respectively), and this distribution can be understood by dividing the characters into three categories. First, all characters traditionally associated with the current classification (e.g., shape of anterior commissure, shell ornamentation, nature of deltidial plates, etc.) of Rhynchonellida (Savage et al. 2002; Manceñido et al. 2007; Savage 2007) are broadly inconsistent with the combined analysis (Fig. 8a). Second, a large subset of characters (e.g., type of shell mineralization; calcareous lophophore supports) with high consistency are those associated with the division between the articulated in-group taxa and the inarticulated out-group (Fig. 8b), and do not vary within the Rhynchonellida, and are thus uninformative with respect to relationships among rhynchonellides. The third category (Fig. 8c), which includes the remainder of the characters used, is mainly inconsistent as measured against the combined analysis, with a composite consistency index of 0.399. The few high-consistency characters in the remainder are those with missing data, which artificially inflates their measured consistency. Thus, it appears that we lack any morphological characters that strongly support the groupings inferred within Rhynchonellida classification, if we accept the Bayesian combined analyses (or the molecular-only analyses; Cohen and Bitner 2013a.; Bitner and Cohen 2015).

This incongruence may be explained by multiple possibilities or any combination thereof. Morphological data associated with rhynchonellide skeletal elements may simply be too homoplastic to resolve relationships among extant lineages. Morphological features we generally recognize from rhynchonellide anatomy might be better indicators of ecological or functional constraints than we currently realize, in which case, morphology-based higher taxa in the Rhynchonellida may not constitute evolutionary units: that is, they may not even be paraphyletic groupings that reflect some record of relationships. Instead, the use of such higher taxa in macroevolutionary studies may instead be capturing patterns of ecological or environmentally driven change in convergent morphological features, potentially decoupled from the underlying history of diversification. If real, this phenomenon may extend well beyond the Rhynchonellida, given the molecular–morphological incongruence claimed in other articulated brachiopod groups (Bitner and Cohen 2015).

However, crown rhynchonellide lineages may well be anciently diverged, such that their morphology is too homoplastic, and the addition of extinct, morphologically intermediate taxa is needed to extricate relationships (Wiens 2009). Ultimately, the most important step forward to evaluating morphological systematics in this group will be to evaluate morphology in conjunction with as large a sample of extinct, potentially crown-clade rhynchonellide taxa as possible.

### Molecular Phylogenetics in the Rhynchonellida

We should not ignore the possibility that confounding issues do not lie solely with the morphological data. The topology supported by the molecular and combined data may be erroneous, a result of artifacts stemming from the aforementioned missing data issues or other issues uniquely associated with the use of ribosomal DNA in phylogenetics. The SSU 18S rDNA gene may not be the most appropriate gene for a phylogenetic analysis at the genus level (Adoutte and Phillippe 1993; Halanych et al. 1995; 1996; Abouheif et al. 1998; Cohen 2007). Cohen (2007) states that it may not adequately resolve the shallowest branches of the extant brachiopod lineages, since SSU 18S rDNA is a relatively slow-evolving gene and thus may not accumulate enough phylogenetic signal over short evolutionary time spans, making it difficult to infer relationships among young lineages. The original nucleotide signal may also reach saturation, overwriting phylogenetic signal in the ribosomal DNA shared by anciently diverged lineages (Cohen 2007). We find it difficult to reject either scenario, given our lack of strong prior assumptions regarding the age of the rhynchonellide crown clade, nor the ages of divergences within that crown clade.

In this study, we limited ourselves to examining the incongruence via a detailed investigation of the morphological dataset, as testing the stability of the molecular data would require collecting additional molecular data, ideally from a wider selection of genes. This was not feasible for the present study. Working with limited molecular data of unknown quality is simply a reality of research on certain nonvertebrate groups that have been sequenced for a small number of genes, by a small number of researchers. Species in 10 of the 19 extant rhynchonellide genera live today at bathyal or abyssal depths in apparently small populations, making them rare, difficult to collect, and poorly studied in terms of their morphological variation. This very deep habitat might also argue against excessive gene flow among populations, which could facilitate multiple independent origins of various morphological characters, resulting in excessive homoplasy in morphology.

### Implications for Systematics in the Rhynchonellida

Several conclusions about rhynchonellide phylogeny with respect to their classification can be made from our analyses of morphological, molecular and combined datasets (Fig. 5b; data on taxonomic group membership for each of the 40 extant rhynchonellide brachiopod species is given in Supplementary Appendix S5 available on Dryad). First, extant rhynchonellides are monophyletic relative to terebratulide and inarticulated brachiopods, confirming earlier studies (e.g., Cohen and Bitner 2013a.). Adding extinct taxa to these analyses will allow us to develop a much more comprehensive hypothesis of relationships within the Rhynchonellida crown clade and also establish crown and total clade ages and relationships among extinct and extant genera in each clade.

Furthermore, if we accept the results of the Bayesian combined analyses from this study (Fig. 5), they suggest considerable convergence in morphology among genera currently classified in each of the four superfamilies in Rhynchonellida with extant representatives. In the combined analyses, Norelloidea (Ager 1959) is a basal, paraphyletic superfamily. Each clade in these combined analyses includes, as a stem-ward member, a genus classified currently in Norelloidea. *Parasphenarina* and *Manithyris* are more distantly related than is indicated by their classification in the same subfamily (Hispanirhynchiinae in Frieleiidae). *Neorhynchia* and *Frieleia* are more closely related than indicated by their classification in different subfamilies in Frieleiidae. *Tethyrhynchia*, currently classified in a monogeneric family, is more closely related to *Parasphenarina* than to the other norelloid genera. Presently, *Acanthobasiliola* is placed in its own subfamily in the family Basiliolidae, but in the Bayesian combined analyses is much more distantly related to the other extant basiliolids in Pugnacoidea compared with other extant rhynchonellides. *Hemithiris* and *Notosaria* are classified in separate families within the same superfamily (Hemithiridoidea), but these genera are distantly related to one another in the Bayesian combined analyses. Some of these discrepancies between the traditional systematic classification, and the results of our combined dataset analyses, imply that small body size may be less evolutionarily labile than previously thought, as both *Tethyrhynchia* and *Parasphenarina* have very small (less than 1 mm) body sizes as adults. Conversely, there may be relatively more convergence toward small body sizes, as both *Hemithiris* and *Notosaria* have relatively large body sizes for rhynchonellides as adults (more than 20 mm).

Finally, the position of *Cryptopora* is unstable across the analyses we have performed, including the Bayesian combined analyses. This is consistent with instability in its classification as well (Manceñido et al. 2002). Together with *Aulites* and an extinct genus, it is placed in Cryptoporidae (Muir-Wood 1955) with the note “a seemingly primitive and controversial group that is tentatively included in this superfamily [Dimerelloidea] in spite of a considerable Cretaceous gap; alternatively, possible relationships with norelloids may not be totally ruled out.” (Manceñido et al. 2002). This is despite the fact that Norelloidea itself is a basal, paraphyletic superfamily.

### Implications for Systematic Paleontology as an Endeavor

If morphology may not always be a trustworthy indicator of phylogenetic relationships, paleontologists who depend heavily on morphology for inferring phylogenetic relationships must develop approaches to identify misleading circumstances, and correct for them, if and when possible. In this case, we chose to attempt such an investigation by comparing independent molecular and morphological datasets for the same set of extant taxa, from a group with a rich and well-studied fossil record. What we found was that the molecular and morphological datasets were exceedingly discordant and that combined analysis only seemed to be consistent when i) Bayesian methods were used and ii) molecular data alone were analyzed. No third alternative topology was found by combining the data, unlike some other combined analyses (e.g., Wiens et al. 2010). We do not necessarily accept from this evidence that the molecular signal is “true” and the morphological signal is “false.” There are clear concerns about both datasets, and the results of our sensitivity analyses suggest that we cannot reject that the topology inferred is not a function of data partition size. Only the collecting of further datasets, such as additional sequence data from other genes, and additional morphological data from extinct taxa, will allow us to test whether the present molecular dataset is sufficient to reconstruct robust phylogenetic hypotheses. Several taxa with substantial missing rDNA sequence data appear to attract one another in the molecular analyses, and this suspicious behavior warrants skepticism. However, it is important to point out that members of the extant Rhynchonellida are generally uncommon marine invertebrates: their collection, sampling, and further sequencing will not be an easy task. Further collection of sequence data will be unlikely to occur without specifically targeted research initiatives, such as those aimed at better understanding deep marine faunas or brachiopod phylogenomics.

At the moment though, the weight of evidence does imply that the morphological data may be more problematic than the molecular data. As noted, both the Bayesian and maximum parsimony combined analyses are more congruent with molecular-only analyses, than with morphology-only analyses, although our sensitivity analyses suggest that this congruence may reflect the size difference between the molecular and morphological character partitions. Results were contradictory across multiple morphology-only analyses, and the placement of taxa without molecular data was inconsistent among those combined analyses that included all 26 taxa. The differences among the morphological analyses may be the best possible indicator of poor signal in morphological data, and thus a sign of caution when an independent molecular dataset isn’t available, as is the case when studying extinct taxa. When morphological datasets are sensitive to small changes in either the data included or the analyses performed, paleontologists would be wise to use caution in their interpretation of the phylogenetic relationships inferred. Alternatively, it is possible that including a large number of extinct, potential morphological intermediates from the fossil record could rescue the morphological data, revealing those phenotypic characters that do carry phylogenetic signal.

## Supplementary Material

Data available from the Dryad Digital Repository: http://dx.doi.org/10.5061/dryad.31048.2
